# Relief of Pain in Uterine Cancer

**Published:** 1877-05

**Authors:** 


Relief of Pain in Uterine Cancer.—Dr. A. E. Aust-Lawrence, Physician to the Bristol General Hospital, writes to the *Medical Times and Gazette*, March 24th:

I have, unfortunately, generally under my care in hospital and private practice, about from twenty to thirty cases of cancer of the uterus, vagina, or rectum; and the experience of the past twelve months has led me to rely, to a great extent, on the following treatment for the relief of pain: In cases of medullary cancer of the uterus, and also of advanced epithelioma in the same region, I have been struck with the marked relief often derived from the administration of ergot, in doses of thirty minims every six hours. There is a relief from the intense throbbing which, as a rule, only subsides with each attack of hemorrhage, which, of course, brings with it great exhaustion. I consider the ergot acts in the ordinary way, by lessening the amount of blood in the uterus; and it may also check, to a slight extent, the rapid breaking down of the affected part. A case of medullary cancer in a young woman, thirty-one years of age, was rendered very much less painful by ergot than by any other remedy which was tried. I have a case now under my care, of sarcoma of the uterus, the pain of which is very much relieved by full doses of ergot.

Another drug I have found of great value is croton-chloral hydrate. This, in my experience, has not very much power to lessen the pain at the seat of the cancer, but it is very valuable in lessening the reflected pains in the back, thighs and groins; and this it has done in several of my cases to a very marked degree. As a local remedy I have found carbolic acid very valuable. I apply it, full strength, by means of a little piece of cotton-wool, through a very small speculum, to the cancerous surface, and then order a lotion with one drachm of the glycerini acidi carbolici to half a pint of water, to be used as an injection night and morning. I have found this drug, used in the way I mention, of great value.

Of course, other drugs suggest themselves to every one, such as opium, Indian hemp, bromide of potassium, etc.; but what I wished to show is that ergot is a very valuable agent in helping to control pain in these cases; that locally I have had better results from carbolic acid than from anything else. I might also add that a very valuable way of relieving pain in these cases is by small blisters in the groins, dressed with an ointment containing morphia.

